# Presepsin and platelet to lymphocyte ratio predict the progression of septic subclinical acute kidney injury to septic acute kidney injury: a pilot study

**DOI:** 10.1186/s13104-022-06103-2

**Published:** 2022-06-20

**Authors:** Yuichiro Shimoyama, Osamu Umegaki, Noriko Kadono, Toshiaki Minami

**Affiliations:** 1 Department of Anesthesiology, Intensive Care Unit, Osaka Medical and Pharmaceutical University Hospital, Osaka Medical and Pharmaceutical University, 2-7 Daigaku-machi, Takatsuki, Osaka 569-8686 Japan; 2 Department of Anesthesiology, Osaka Medical and Pharmaceutical University Hospital, Osaka Medical and Pharmaceutical University, Takatsuki, Osaka 569-8686 Japan

**Keywords:** Presepsin, Subclinical acute kidney injury, Sepsis, Sepsis-3 definition, Inflammation-based prognostic scores

## Abstract

**Objective:**

This study aimed to determine whether presepsin and inflammation-based prognostic scores can predict the progression of septic subclinical acute kidney injury (AKI) to septic AKI among intensive care unit (ICU) patients.

**Results:**

Presepsin values were measured immediately after ICU admission (baseline) and on Days 2, 3, and 5 after ICU admission. Glasgow Prognostic Score, neutrophil to lymphocyte ratio, platelet to lymphocyte ratio (PLR), Prognostic Index, and Prognostic Nutritional Index were measured at baseline. Presepsin values and these indices were compared between septic AKI and septic subclinical AKI patients. There were 38 septic AKI patients and 21 septic subclinical AKI patients. Receiver operating characteristic curve analyses revealed the following cut-off values for AKI (relative to subclinical AKI): 708.0 (pg/ml) for presepsin on Day 1 (AUC, 0.69; sensitivity, 82%; specificity, 52%), 1283.0 (pg/ml) for presepsin on Day 2 (AUC, 0.69; sensitivity, 55%; specificity, 80%), and 368.66 for PLR (AUC, 0.67; sensitivity, 71%; specificity, 62%). Multivariate logistic regression analyses revealed PLR to be a predictor of septic subclinical AKI (odds ratio, 1.0023; 95% confidence interval, 1.0000–1.0046; p = 0.046). Presepsin and PLR predicted the progression of septic subclinical AKI to septic AKI and the prognosis of subclinical septic AKI patients.

**Supplementary Information:**

The online version contains supplementary material available at 10.1186/s13104-022-06103-2.

## Introduction

Sepsis is the main cause of mortality in critically ill intensive care unit (ICU) patients [1] and the most common cause of acute kidney injury (AKI) among critically ill patients [2]. About 20–30% of hospitalized patients are affected with AKI, which is independently associated with elevated mortality [3, 4]. The identification of neutrophil gelatinase-associated lipocalin (NGAL) as a relevant protein in the disease allowed for the creation of a new category of AKI referred to as “subclinical AKI”. Subclinical AKI is a condition in which a patient has elevated NGAL levels, but no signs of clinical AKI (i.e., increased serum creatinine and/or a decreased urinary output). Subclinical AKI is associated with a greater risk of adverse outcomes, including death and renal replacement therapy [5].

Presepsin is a subtype of soluble CD14 (CD14-ST) [6]. It has a higher specificity for diagnosing sepsis than procalcitonin (PCT) and IL-6 [7], and thus has been proposed to be useful for assessing the prognosis and monitoring the course of sepsis [8]. Another advantage of presepsin is that it can be measured in less than 17 min with a compact fully-automated immunoanalyzer (PATHFAST®; Mitsubishi Chemical Medience Corporation, Tokyo, Japan) [9]. The Glasgow Prognostic Score (GPS; calculated based on serum C-reactive protein (CRP) and albumin levels), neutrophil to lymphocyte ratio (NLR), platelet to lymphocyte ratio (PLR), Prognostic Nutritional Index (PNI; calculated based on albumin and lymphocyte counts), and the Prognostic Index (PI; calculated based on serum CRP and white blood cell counts) are inflammation-based prognostic scores which are useful prognostic biomarkers for many types of cancer [10].

No study to date has investigated the association of presepsin values (alone or in combination with the above-mentioned inflammation-based prognostic scores) with the progression of septic subclinical AKI to septic clinical AKI and the prognosis of septic subclinical AKI among ICU patients. To this end, the present study aimed to prove the following hypotheses: (1) presepsin predicts the progression of septic subclinical AKI to septic AKI, and prognosis of septic subclinical AKI, among ICU patients; and (2) the ability of presepsin to predict the above is superior to inflammation-based prognostic scores and can be improved when combined with inflammation-based prognostic scores.

## Main text

### Methods

#### Patients and study design

This single-center prospective study was conducted in a 16-bed ICU. The study design, handling of informed consent and patient inclusion, and definition of “inflammation-presepsin scores [iPS]” used in the present study were described previously [11]. The study protocol was approved by the Ethics Committee of Osaka Medical College (Osaka, Japan), and was carried out in accordance with The Code of Ethics of the World Medical Association (Declaration of Helsinki). In total, 61 adult patients aged ≥ 18 years who were diagnosed with sepsis according to the Sepsis-3 definition (1) and admitted to the ICU were prospectively examined from December 2017 to August 2019. For category classification, total scores were calculated (hereafter, ‘‘inflammation-presepsin scores [iPS]’’) as follows: a score of 1 was assigned if the presepsin value and inflammation-based prognostic scores at baseline were above cutoffs determined by receiver operating characteristic (ROC) curve analysis for 28-day mortality; a score of 0 was assigned if they were below the cutoffs (total score range, 0–2 points).

Septic AKI was defined as stage ≥ 1 kidney disease according to the Kidney Disease: Improving Global Outcomes (KDIGO) classification [12]. Septic subclinical AKI is not encompassed by the definition of septic AKI above, and is defined as having urinary NGAL levels greater than the upper normal limit (> 30.5 ng/ml) in the absence of diagnostic increases in serum creatinine. GPS, NLR, PLR, PI, and PNI were examined at baseline. Presepsin values, inflammation-based prognostic scores, iPS, and changes (Δ) in presepsin values relative to baseline values at each sampling point were compared between patients with septic AKI and septic subclinical AKI, and between surviving and non-surviving septic AKI patients or septic subclinical AKI patients.

#### Laboratory assessments

Presepsin concentration was measured by PATHFAST^®^ (Mitsubishi Chemical Medience Corporation, Tokyo, Japan) [9]. Threshold values were as follows: (a) 300–500 pg/ml: “systemic infection (sepsis) possible”; (b) 500–1000 pg/ml: “significant risk of systemic infection progression (severe sepsis); increased risk of unfavorable outcome”; and (c) ≥ 1000 pg/ml: “high risk of systemic infection progression (severe sepsis/septic shock); high risk for mortality after 30 days comparable with a Sequential Organ Failure Assessment (SOFA) score ≥ 8” [13, 14].

### Statistical analysis

Categorical data are reported as percentages and compared using Fisher’s exact test. Continuous data are reported as medians with inter-quartile ranges and compared using the Mann–Whitney U test. ROC curves were generated for presepsin values, inflammation-based prognostic scores, iPS, and Δpresepsin, and areas under the curve (AUCs), cut-off values, sensitivities, and specificities were calculated. For presepsin on Day 1, Kaplan–Meier curves were constructed for each mortality category, and the log-rank test was performed. Presepsin values, inflammation-based prognostic scores, iPS, Δpresepsin, SOFA, and quick SOFA (qSOFA) (variables with p < 0.05 in univariate analysis) were examined further by multivariate logistic regression analyses for predicting septic subclinical AKI compared to septic AKI. The objective variables were septic subclinical AKI (value of 1) and septic AKI (value of 0). P < 0.05 was considered statistically significant. JMP software version 11.00 (SAS Institute Inc., NC, USA) was used for all statistical analyses.

### Results

Baseline characteristics of 61 adult patients included in the present study are shown in Table 1. Median age was 75 years (range: 66.0–81.0) in septic AKI patients and 75 years (range: 69.0–78.0) in septic subclinical AKI patients. No significant differences were observed in age and sex between septic AKI patients and septic subclinical AKI patients (Table 1).

**Table 1:** Baseline demographic characteristics

Variable	AKI (n = 38)		Subclinical AKI (n = 21)	*p* value
Age (years)	75.0 (66.0–81.0)		75.0 (69.0–78.0)	0.769
Sex (male) (%)	24.0 (63.2)		14.0 (66.7)	1.000
Cancer (%)	21.0 (55.3)		12.0 (57.1)	1.000
Coronary artery disease (%)	1.0 (2.6)		1.0 (4.8)	1.000
Diabetes mellitus (%)	9.0 (23.7)		1.0 (4.8)	0.157
Hypertension (%)	15.0 (39.5)		2.0 (9.5)	0.018
Albumin (g/dL)	2.3 (1.8–2.9)		2.3 (2.0–3.1)	0.663
CRP (mg/dL)	14.1 (7.7–20.1)		9.3 (2.6–13.2)	0.037
WBC (× 10^9^ l^−1^)	11.6 (5.9–17.5)		9.9 (5.4–14.6)	0.406
Neutrophil count (× 10^9^ l^−1^)	9.2 (4.2–15.4)		7.2 (1.3–13.5)	0.457
Lymphocyte count (× 10^9^ l^−1^)	505.0 (333.5–926.5)		512.0 (219.0–610.0)	0.358
Plt count (× 10^4^ mm^−3^)	150.0 (110.3–241.8)		182.0 (126.0–241.0)	0.311
Fibrinogen (mg/dL)	592.5 (348.0–682.8)		613.5 (447.0–736.8)	0.556
Survival (dead) (%)	16.0 (42.1)		3.0 (14.3)	0.041
ARDS (%)	10.0 (26.3)		1.0 (4.8)	0.077
Shock (%)	26.0 (68.4)		11.0 (52.4)	0.268
DIC (%)	22.0 (57.9)		4.0 (19.0)	0.006
Presepsin on Day 1 (pg/mL)	1262.0 (752.0–2078.5)		707.0 (306.0–1536.0)	0.019
Presepsin on Day 2 (ng/mL)	1333.0 (671.0–2433.0)		797.5 (345.0–1227.5)	0.038
Presepsin on Day 3 (ng/mL)	993.0 (563.0–2123.0)		813.0 (478.0–1827.0)	0.348
Presepsin on Day 5 (ng/mL)	1313.0 (689.0–2759.0)		711.0 (410.0–1055.0)	0.062
ΔPresepsin Day 2—Day 1 (pg/mL)	− 65.00 (− 314.0 to 507.0)		6.5 (− 215.0 to 124.5)	0.959
ΔPresepsin Day 3—Day 1 (pg/mL)	− 236.00 (− 872.0 to 390.0)		− 39.0 (− 723.0 to 12.0)	0.790
ΔPresepsin Day 5—Day 1 (pg/mL)	− 323.50 (− 1547.5 to 719.5)		− 366.5 (− 762.3 to 2.8)	1.000
NGAL on Day 1 (ng/mL)	506.50 (194.7–2562.0)		132.0 (54.6–277.8)	P < 0.001
NGAL on Day 2 (ng/mL)	851.20 (332.4–1496.2)		140.4 (66.8–271.0)	P < 0.001
NGAL on Day 3 (ng/mL)	487.30 (225.5–1212.4)		87.2 (57.4–161.1)	0.003
NGAL on Day 4 (ng/mL)	630.50 (84.9–1272.7)		74.8 (30.4–137.6)	0.013
GPS	2.0 (1.0–2.0)		1.0 (1.0–2.0)	0.332
iPS–GPS	1.0 (1.0–1.0)		1.0 (0–1.0)	0.134
NLR	13.1 (4.3–34.8)		16.3 (8.9–27.1)	0.496
iPS–NLR	1.0 (0–2.0)		1.0 (1.0–1.0)	0.643
PLR	283.2 (170.3–387.0)		401.5 (243.8–855.7)	0.033
iPS–PLR	1.0 (0–1.0)		1.0 (1.0–1.0)	0.585
PI	1.0 (1.0–2.0)		1.0 (0–2.0)	0.281
iPS–PI	1.0 (0–1.0)		1.0 (0–1.0)	0.263
PNI	27.3 (21.3–33.6)		23.3 (16.7–29.5)	0.254
iPS–PNI	1.0 (1.0–2.0)		1.0 (1.0–1.0)	0.119
SOFA	9.5 (7.0–11.0)		9.0 (7.0–11.0)	0.930
qSOFA	2.0 (1.0–3.0)		2.0 (1.0–3.0)	0.599

*AKI* acute kidney injury, *CRP* C-reactive protein, *WBC* white blood cell, *ARDS* acute respiratory distress syndrome, *DIC* disseminated intravascular coagulation, *NGAL* neutrophil gelatinase-associated lipocalin, *GPS* Glasgow Prognostic Score, *iPS* inflammation-presepsin score, *NLR* neutrophil to lymphocyte ratio, *PLR* platelet to lymphocyte ratio, *PI* Prognostic Index, *PNI* Prognostic Nutritional Index, *SOFA* Sequential Organ Failure Assessment, *qSOFA* quick Sequential Organ Failure Assessment

Among the 61 patients, 38 (62.3%) were diagnosed with septic AKI as defined as stage ≥ 1 kidney disease using the KDIGO classification, 21 (34.4%) were diagnosed with septic subclinical AKI, and two were diagnosed with neither septic AKI nor septic subclinical AKI. Six septic AKI patients were initiated on renal replacement therapy. One patient had been withdrawn from renal replacement therapy, one patient progressed to end-stage renal disease, and four patients died in the ICU. ROC curve analyses revealed the following cut-off values (Table 2) for AKI (relative to septic subclinical AKI): 708.0 (pg/ml) for presepsin on Day 1 (AUC, 0.69; sensitivity, 82%; specificity, 52%), 1283.0 (pg/ml) for presepsin on Day 2 (AUC, 0.69; sensitivity, 55%; specificity, 80%), and 368.66 for PLR (AUC, 0.67; sensitivity, 71%; specificity, 62%).

**Table 2:** Receiver operating characteristic curve analysis for predicting septic subclinical AKI to septic AKI progression

Variable	AUC	Cut-off	P value	Sensitivity (%)	Specificity (%)
Presepsin on Day 1 (pg/mL)	0.69	708.00	0.02	0.82	0.52
Presepsin on Day 2 (pg/mL)	0.69	1283.00	0.03	0.55	0.80
Presepsin on Day 3 (pg/mL)	0.60	872.00	0.35	0.57	0.64
Presepsin on Day 5 (pg/mL)	0.72	1262.00	0.03	0.59	0.89
ΔPresepsin Day 2—Day 1 (pg/mL)	0.50	13.00	0.96	0.66	0.50
ΔPresepsin Day 3—Day 1 (pg/mL)	0.53	− 228.00	0.79	0.57	0.62
ΔPresepsin Day 5—Day 1 (pg/mL)	0.50	− 423.00	1.00	0.61	0.50
GPS	0.57	2.00	0.34	0.55	0.57
iPS-GPS	0.61	1.00	0.13	0.76	0.43
NLR	0.55	8.89	0.48	0.45	0.76
iPS-NLR	0.53	2.00	0.63	0.29	0.86
PLR	0.67	368.66	0.03	0.71	0.62
iPS-PLR	0.54	0.00	0.58	0.34	0.76
PI	0.58	1.00	0.31	0.82	0.38
iPS-PI	0.58	1.00	0.26	0.66	0.48
PNI	0.60	26.08	0.27	0.58	0.67
iPS-PNI	0.61	2.00	0.06	0.29	1.00

*AKI* acute kidney injury, *AUC* area under the curve, *GPS* Glasgow Prognostic Score, *iPS* inflammation-presepsin score, *NLR* neutrophil to lymphocyte ratio, *PLR* platelet to lymphocyte ratio, *PI* Prognostic Index, *PNI* Prognostic Nutritional Index

The results of ROC curve analyses and the log-rank test for the prognosis of septic AKI patients and subclinical AKI patients are shown in Table 3; Additional file 1: Table S1, respectively. ROC curve analyses indicated the following cut-off values: 28-day mortality: 1373.0 (pg/ml) for presepsin on Day 1 in septic AKI patients (AUC, 0.78; sensitivity, 83%; specificity, 77%); 60-day mortality: 1373.0 (pg/ml) for presepsin on Day 1 in septic AKI patients (AUC, 0.72; sensitivity, 77%; specificity, 76%) and 1152.0 (pg/ml) in septic subclinical AKI patients (AUC, 0.72; sensitivity, 100%; specificity, 61%); 90-day mortality: 1373.0 (pg/ml) for presepsin on Day 1 in septic AKI patients (AUC, 0.67; sensitivity, 67%; specificity, 74%) and 1152.0 (pg/ml) in septic subclinical AKI patients (AUC, 0.72; sensitivity, 100%; specificity, 61%); 180-day mortality: 1336.0 (pg/ml) for presepsin on Day 1 in septic AKI patients (AUC, 0.67; sensitivity, 69%; specificity, 68%) and 1152.0 (pg/ml) in septic subclinical AKI patients (AUC, 0.72; sensitivity, 100%; specificity, 61%) (Table 3). In the log-rank test, presepsin on Day 1 in septic AKI patients was a significant predictor of 28-day mortality (p = 0.002), 60-day mortality (p = 0.004), 90-day mortality (p = 0.032), and 180-day mortality (p = 0.050) (Additional file 1: Table S1). Multivariate logistic regression analyses revealed PLR to be a significant predictor of septic subclinical AKI (OR, 1.0023; 95% CI, 1.0000–1.0046; p = 0.046) (Additional file 2: Table S2).

**Table 3:** Receiver operating characteristic curve analysis of presepsin on Day 1

Variable	AUC	Cut-off	P value	Sensitivity (%)	Specificity (%)
28-day mortality					
AKI	0.78	1373.00	0.00	0.83	0.77
Subclinical AKI					
60-day mortality					
AKI	0.72	1373.00	0.03	0.77	0.76
Subclinical AKI	0.72	1152.00	0.05	1.00	0.61
90-day mortality					
AKI	0.67	1373.00	0.08	0.67	0.74
Subclinical AKI	0.72	1152.00	0.05	1.00	0.61
180-day mortality					
AKI	0.67	1336.00	0.07	0.69	0.68
Subclinical AKI	0.72	1152.00	0.05	1.00	0.61

*AUC* area under the curve, *AKI* acute kidney injury

### Discussion

There were 38 septic AKI patients in the present study population. Among 23 non-septic AKI patients, 21 (91.3%) were diagnosed with septic subclinical AKI (Table 1). The proportion of patients with septic subclinical AKI among non-septic AKI patients was larger than expected. Presepsin and PLR predicted the progression of septic subclinical AKI to septic AKI. In particular, the AUC, sensitivity, and specificity of presepsin on Day 1 for predicting progression were 0.69, 82%, and 52%, respectively, corresponding to a higher sensitivity than those observed for presepsin on Days 2, 3, and 5, as well as PLR (Table 2). These results demonstrate that presepsin on Day 1 is a good “rule out” test for predicting the progression of septic subclinical AKI to septic AKI.

Our results will contribute to the prevention and management of progression of septic subclinical AKI to septic AKI. Septic AKI is associated with poor patient prognoses, including death [5]. Serum creatinine is a delayed, low-sensitivity, potentially misleading biomarker of AKI [15, 16], as it is influenced by many confounding factors [17] and thus may fail to detect the progression of septic subclinical AKI to septic AKI in septic patients who are at increased risk of death. Presepsin, on the other hand, was found to predict the progression of septic subclinical AKI to septic AKI in the absence of diagnostic increases in serum creatinine (Table 2). Thus, elevated presepsin might help lead to earlier diagnosis and rapid introduction of conventional interventions to prevent the progression of septic subclinical AKI to septic AKI. The use of elevated presepsin as a predictor may thus increase the likelihood that a treatment will be successful, since changes in its levels occur rapidly and can be measured quickly (in less than 17 min), whereas it can take hours to detect/measure changes in NGAL and days for serum creatinine.

Presepsin cut-off values indicated in the present study for predicting the progression of septic subclinical AKI to septic AKI (Table 2), and prognosis in septic subclinical AKI patients (Table 3), were higher than those previously reported to predict severe sepsis and septic shock [13, 14]. Nakamura et al. reported a significant negative correlation between presepsin levels and estimated glomerular filtration rate in both non-sepsis and sepsis patients [18]. Presepsin values were significantly elevated in ICU patients with renal failure and end-stage kidney disease, irrespective of whether they had sepsis or not. Presepsin values in patients with sepsis ranged from 2,632 to 20,000 pg/ml, while those in patients without sepsis ranged from 2,134 to 19,633 pg/ml [18]; the presepsin cut-off values identified in the present study were lower than these. Our results demonstrate that a lower presepsin cut-off value should be adopted for predicting the progression of septic subclinical AKI to septic AKI compared to that used for ICU patients with renal failure and end-stage kidney disease.

The AUC, sensitivity, and specificity of PLR for predicting the progression of septic subclinical acute kidney injury to septic AKI were 0.67, 71%, and 62%, respectively (Table 2), and PLR was demonstrated to be a predictor of septic subclinical AKI in multivariate logistic regression analyses (Additional file 2: Table S2). Previously, Smith et al. reported that PLR is a significant prognostic marker in pancreatic cancer patients [19]. PLR was suggested to be useful for predicting the progression of septic subclinical AKI to septic AKI in the present study. PLR can be obtained at low cost and rapidly, and is convenient for bedside use. In small- and medium-sized hospitals where presepsin values cannot be easily measured, PLR provides information for aggressive and supportive therapy in septic subclinical AKI patients within the first few hours of ICU admission. Among the various variables which can be tested to assess inflammation, platelet and lymphocyte counts (which are used to calculate PLR) are important for predicting the progression of septic subclinical AKI to septic AKI.

### Conclusions

Presepsin and PLR predicted the progression of septic subclinical AKI to septic AKI, as well as the prognosis of subclinical septic AKI patients. In particular, our findings suggest that presepsin on Day 1 may serve as an easy “rule out” test for predicting the progression of septic subclinical AKI to septic AKI. Further studies aimed at understanding the exact role of presepsin and PLR in predicting disease progression, and prognosis of subclinical septic AKI patients, are warranted.

### Limitations

This study has several limitations. First, the present study was conducted at a single center ICU with a small sample size. Second, we used only a single biomarker, and no comparisons were made with other biomarkers.

## Supplementary Information

**Additional file 1: Table S1**. Log-rank test of presepsin on Day 1.

**Additional file 2:** **Table S2**. Multivariate logistic regression analysis for prediction of septic subclinical AKI.

## Data Availability

The datasets used and/or analyzed during the current study are available from the corresponding author on reasonable request.

## Abbreviations

ICU: Intensive care unit
AKI: Acute kidney injury
NGAL: Neutrophil gelatinase-associated lipocalin
KDIGO: Kidney Disease: Improving Global Outcomes
GPS: Glasgow Prognostic Score
CRP: C-reactive protein
NLR: Neutrophil to lymphocyte ratio
PLR: Platelet to lymphocyte ratio
PNI: Prognostic Nutritional Index
PI: Prognostic Index
SOFA: Sequential Organ Failure Assessment
qSOFA: Quick Sequential Organ Failure Assessment
ROC: Receiver operating characteristic
AUC: Area under the curve
