# Supplementary material for: Presepsin and platelet to lymphocyte ratio predict the progression of septic subclinical acute kidney injury to septic acute kidney injury: a pilot study
Source: BMC Res Notes. 2022 Jun 20;15:212. doi: 10.1186/s13104-022-06103-2 (PMC9208238; doi:10.1186/s13104-022-06103-2)
Supplement: Supplementary file 1 [file 13104_2022_6103_MOESM1_ESM.docx]

Table S1. Log-rank test of presepsin on Day 1

Variable	P value
28-day mortality	
AKI	0.002
subclinical AKI	
60-day mortality	
AKI	0.004
subclinical AKI	0.671
90-day mortality	
AKI	0.032
subclinical AKI	0.671
180-day mortality	
AKI	0.050
subclinical AKI	0.671

AKI, acute kidney injury
