# Supplementary material for: Presepsin and platelet to lymphocyte ratio predict the progression of septic subclinical acute kidney injury to septic acute kidney injury: a pilot study
Source: BMC Res Notes. 2022 Jun 20;15:212. doi: 10.1186/s13104-022-06103-2 (PMC9208238; doi:10.1186/s13104-022-06103-2)
Supplement: Supplementary file 2 [file 13104_2022_6103_MOESM2_ESM.docx]

Variable	Odds ratio	95% CI		*P* value
PLR	1.0023	1.0000	1.0046	0.046
Presepsin on Day 1	1.0002	0.9990	1.0013	0.784
Presepsin on Day 2	0.9997	0.9987	1.0007	0.562

AKI, acute kidney injury; CI, confidence interval; PLR, platelet to lymphocyte ratio
